# Deep brain stimulation creates informational lesion through membrane depolarization in mouse hippocampus

**DOI:** 10.1038/s41467-022-35314-1

**Published:** 2022-12-13

**Authors:** Eric Lowet, Krishnakanth Kondabolu, Samuel Zhou, Rebecca A. Mount, Yangyang Wang, Cara R. Ravasio, Xue Han

**Affiliations:** Boston University, Department of Biomedical Engineering, Boston, MA 02215 USA

**Keywords:** Cellular neuroscience, Neural circuits, Wide-field fluorescence microscopy, Optogenetics

## Abstract

Deep brain stimulation (DBS) is a promising neuromodulation therapy, but the neurophysiological mechanisms of DBS remain unclear. In awake mice, we performed high-speed membrane voltage fluorescence imaging of individual hippocampal CA1 neurons during DBS delivered at 40 Hz or 140 Hz, free of electrical interference. DBS powerfully depolarized somatic membrane potentials without suppressing spike rate, especially at 140 Hz. Further, DBS paced membrane voltage and spike timing at the stimulation frequency and reduced timed spiking output in response to hippocampal network theta-rhythmic (3–12 Hz) activity patterns. To determine whether DBS directly impacts cellular processing of inputs, we optogenetically evoked theta-rhythmic membrane depolarization at the soma. We found that DBS-evoked membrane depolarization was correlated with DBS-mediated suppression of neuronal responses to optogenetic inputs. These results demonstrate that DBS produces powerful membrane depolarization that interferes with the ability of individual neurons to respond to inputs, creating an informational lesion.

## Abstract (web summary)

The neurophysiological mechanisms of deep brain stimulation remain poorly understood. Through fluorescence voltage imaging of individual hippocampal neurons in awake mice, the authors show that deep brain stimulation causes membrane depolarization that impairs a neuron’s ability to respond to intrinsic network activity patterns and optogenetic somatic depolarization, thereby creating an informational lesion.

## Introduction

Deep brain stimulation (DBS) is effective in managing many brain disorders, such as epilepsy, Parkinson’s disease, and essential tremor. DBS directly stimulates brain tissue through chronically implanted electrodes and is increasingly considered a neural circuit-specific therapy. Clinically, DBS is typically delivered at high frequencies of 130–200 Hz, as lower frequency stimulations produce no consistent therapeutic effects^1–7^. High-frequency DBS when targeted to the limbic system, such as the hippocampus and the anterior nucleus of the thalamus, is effective at decreasing seizure frequency for drug-resistant epilepsy^5–7^. Similarly, when targeted to the basal ganglia, including the subthalamic nucleus (STN) and globus pallidus internus (GPi), DBS is effective at managing the motor symptoms of Parkinson’s disease^1–4^. The clinical success of DBS in managing epilepsy^5–7^ and movement disorders^1–4^ led to a growing effort on exploring the therapeutic benefits of DBS in many other neurological and psychiatric conditions. For example, DBS of the fornix, a major pathway of the hippocampus, is actively explored for Alzheimer’s disease^8,9^.

While clinically effective, the cellular and circuit mechanisms underlying the therapeutic effects of high-frequency DBS remain unclear^8,10–14^. DBS therapeutic outcomes and time courses are diverse and depend on the specific disease conditions targeted^15^. Since DBS effect in patients with epilepsy and movement disorders is consistent with a pharmacological lesion or surgical removal of the targeted brain tissue, DBS was first thought to inhibit local neural activity, likely via membrane depolarization-induced action potential blockage^11^ or glia-mediated adenosine release^16,17^. Consistent with this hypothesis, electrophysiological recordings in Parkinsonian patients showed that transient subsecond long high-frequency DBS generally inhibited neuronal spiking in the STN and GPi^14,18^. However, mechanistic studies using biophysical modeling and electrophysiological analysis in animal models and in vitro brain tissue models demonstrated that electrical pulses can directly excite axons leading to antidromic activation of neurons projecting to the stimulated area or orthodromic activation of downstream postsynaptic neurons^16,18^. Indeed, direct axonal activation at the stimulation site could recruit local synaptic transmission and synaptic plasticity that may contribute to the failure of sustained inhibition of neuronal spiking during prolonged seconds-long high-frequency DBS^18^.

An alternative theory is that DBS entrains neural activity^8^, which interferes with individual neuronsʼ ability to respond to synaptic inputs and thereby creates an informational lesion^19^ that disrupts pathological network patterns. The effects of DBS on network dynamics have inspired the exploration of stimulation pulse patterns that may better engage plasticity mechanisms^20,21^, and the development of closed-loop DBS systems that target network-level pathological electrical field features in epilepsy^2,21^ and Parkinson’s disease^5,6,22,23^. Recent long-term clinical studies reported an impressive reduction of seizure frequency by closed-loop responsive neurostimulation that targeted subject-specific abnormal intracranial electroencephalogram patterns in epilepsy patients^24,25^. To characterize the network effect of DBS, Paulk *et al*. mapped the cortical responses evoked by single-pulse electrical stimulation delivered via intracranial electrodes in epilepsy patients and confirmed that DBS evoked broad network responses that were stimulation location and pulse parameter dependent^26^. Finally, using cellular fluorescent calcium imaging techniques, a couple of recent studies in animal models demonstrated that DBS altered the relationship of intracellular calcium dynamics and behavior, supporting a network effect of DBS^27–29^.

Given the intricate connectivity of brain circuits, it is expected that DBS targeted to a particular anatomical structure would engage interconnected brain regions to produce broad network responses. But the fact that DBS therapeutic effect is stimulation pulse pattern dependent even for the same brain target underscores the importance of generating relevant network responses for a particular disease condition. Thus far, electrophysiological studies have focused on analyzing the effect of DBS on individual neuronsʼ spike rate and the network-level population electrical field potentials. It is unknown how DBS alters individual neuronsʼ cellular voltage dynamics that determine spike production and ultimately the network effects of DBS. Specifically, it is unclear how DBS alters the subthreshold membrane voltage in individual neurons, which influences the ability of individual neurons to process synaptic inputs and to transform synaptic inputs to supra-threshold spiking output. To answer this question, we performed cellular voltage imaging using the recently developed fully genetically encoded soma-targeted  voltage sensor SomArchon^30^, free of electrical stimulation artifacts. SomArchon exhibits rapid voltage responses with a rising time constant of 0.6 ms and a decay time constant of 1.1 ms, and can reliably track individual action potentials^30,31^. Using SomArchon, we measured both the subthreshold membrane voltage dynamics and the action potentials of individual hippocampal CA1 neurons in awake mice, while delivering either high-frequency clinically effective 140 Hz DBS ^32,33^ or the less effective 40 Hz DBS. Further, to understand how the cellular effects of DBS influence neuronal responses to inputs, we tested the ability of individual neurons in responding to depolarizing voltage inputs evoked by somatic optogenetics with or without DBS.

## Results

### Single-cell SomArchon fluorescence imaging enables electrical artifact-free cellular membrane voltage recordings during DBS

**Fig. 1:**
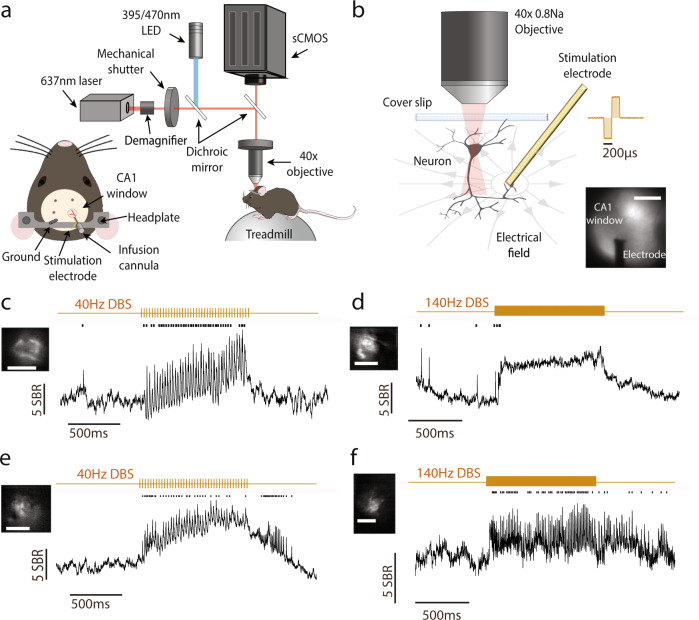
Single-cell SomArchon fluorescence voltage imaging enables artifact-free neural recordings during DBS. **a** Illustration of the experimental setup, the optical imaging window, and the animal preparation. The schematic was adapted from Piatkevich et al. 2019. **b** Schematic representation of a recorded CA1 neuron in the electric field generated by the electrode a few hundred microns away, and an example empirically measured electrical stimulation waveform. Lower right is an example imaging field showing GFP fluorescence from neurons expressing SomArchon-GFP and CoChR and the shadow of the nearby electrode. Scale bar 500 µm. The schematic neuron was adapted from Scidraw (doi.org/10.5281/zenodo.3925905). **c** Example SomArchon fluorescence before, during and after 40 Hz DBS. Average SomArchon fluorescence of an example neuron is shown in the upper left image. Scale bar, 15 µm. SomArchon trace is shown in black, detected spikes are marked by black ticks and electrical stimulation pulse patterns are in gold. SBR is the spike-to-baseline ratio, defined as the average spike amplitude divided by the standard deviation of the subthreshold SomArchon trace fluctuation for each recorded neuron (see methods). **d** Same as (**c**), but for 140 Hz DBS. **e-f** Same as (**c-d**), but other single neuron trial examples.

To probe the membrane voltage effect of DBS on individual neurons in real-time, we performed voltage imaging of CA1 neurons, through chronically implanted optical imaging windows in awake mice^30^. AAV9-Syn-SomArchon-p2A-CoChR-Kv2.1 was infused through an infusion cannula coupled to the imaging window to express in the same neuron both the near-infrared genetically encoded voltage indicator SomArchon and the blue-light activated channelrhodopsin CoChR^30^. SomArchon fluorescence tracks membrane depolarization with sub-millisecond precision and is insensitive to the blue light needed for CoChR optogenetic activation^30^. SomArchon-expressing neurons were imaged using a custom widefield microscope equipped with a 40X objective lens (NA = 0.8), a 637 nm red laser for fluorescence excitation through a 620/60 nm filter, and a high-speed sCMOS camera that collected the near-infrared SomArchon fluorescence emission through a 706/95 nm filter at 828 Hz. The neurons imaged were about 50–150 µm below the imaging window surface corresponding to CA1 pyramidal layer and stratum oriens. DBS was delivered via a stimulation electrode coupled to the side of the imaging window, with the electrode tip (127 µm in diameter) positioned ~200 µm below the imaging plane, ~0.2–2 mm from the recorded neurons. A skull screw over the cerebellum was used as the ground (Fig. 1a, b). We delivered DBS at 40 Hz or 140 Hz through the stimulation electrode, while performing SomArchon voltage imaging in head-fixed mice navigating on a spherical treadmill. DBS was delivered for 1 second, every 12 seconds, with each biphasic pulse being 400 µs in total width.

To study the effect of DBS on the membrane voltage of individual neurons, we manually segmented the imaged neurons and extracted the SomArchon fluorescence trace at the cell body. Because of the variation in the spatial distance of the implanted stimulation electrode to the optical imaging plane across mice, we first tested a few neurons in each mouse to determine the effective DBS amplitude range for a given mouse. Briefly, we delivered DBS pulses over a small number of test trials with increasing amplitude starting from 10 uA, with 5–10 uA increment, until we saw obvious changes in the spectrogram of the recorded SomArchon fluorescence trace. Spectrogram changes could result from membrane depolarization, hyperpolarization, voltage entrainment, or spike firing rate change. During subsequent recording sessions, we started with testing the lowest current amplitude for a given mouse, and if the lowest amplitude DBS produced no detectable changes in SomArchon trace spectrogram, we incrementally increased current amplitude by 5–10 uA in subsequent trials until a spectrogram response was detected. We then applied the same current amplitude over multiple trials during both 40 Hz and 140 Hz DBS conditions. Across all neurons, the peak amplitude ranged 10–60 µA (38.3 ± 11.4 µA, mean ± standard deviation, *n* = 83 neurons in 7 mice, corresponding to ~10–64 µC/cm^2^ charge density per stimulation phase^34^). For a few neurons, only one of the DBS conditions was available. Using the chosen DBS amplitude, delivered at 40 Hz and 140 Hz, most of the neurons showed a significant increase in SomArchon fluorescence, corresponding to somatic membrane voltage depolarization (Fig. 1c–f, Supplementary Fig. 1). To characterize how DBS selectively impacts action potentials versus subthreshold membrane voltage (Vm), we first identified spikes from the recorded SomArchon traces as time points having fluorescence values greater than 4 standard deviations of the SomArchon trace variations across the entire recording session (detailed in Methods, Supplementary Fig. 3^35^). We then removed the identified spikes from the SomArchon traces and computed the Vm by normalizing the spike-removed SomArchon traces to the mean spike amplitude of the neuron. The slight variation of the DBS amplitude across neurons did not correlate with DBS-induced Vm depolarization (Pearson correlation, *r* = 0.032, *p* = 0.77, *n* = 83, Supplementary Fig. 2).

### DBS induces powerful membrane depolarization without suppressing spike rate at both 40 Hz and 140 Hz, and 140 Hz DBS evokes stronger membrane depolarization than 40 Hz DBS

**Fig. 2:**
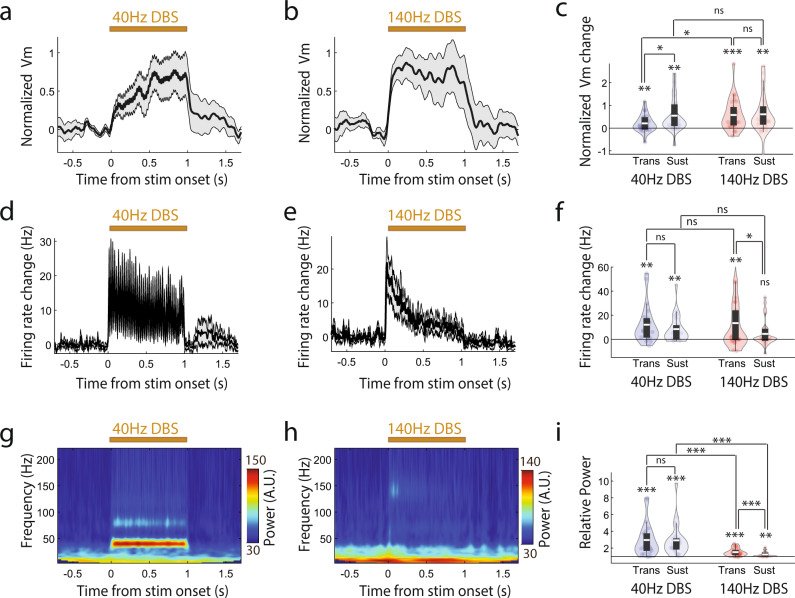
DBS induces powerful membrane depolarization at both 40 Hz and 140 Hz. **a-c** Amplitude of the subthreshold membrane potential (Vm) and corresponding quantifications. **a, b** Population-averaged Vm during 40 Hz DBS (**a**, *n* = 22 neurons) and 140 Hz DBS (**b**, *n* = 26 neurons). Vm is calculated as the fluorescence at each time point divided by the averaged amplitude of all spikes detected in a recording session for a given neuron. DBS-induced Vm changes were computed as normalized Vm by subtracting the mean of the Vm during the pre-stimulation baseline period. Shaded gray area is the standard error of the mean (SEM). **c** Quantification of Vm change from baseline during the transient (0–0.15 s) and sustained (0.15–1 s) periods of 40 Hz and 140 Hz DBS. Data are visualized as violin plots with the outer shape representing the data kernel density and box plots showing interquartile range (1x, 1.5x). The white lines in the boxes are the mean. For 40 Hz DBS, paired t-test, df = 21 neurons, transient: *p* = 0.002; sustained: *p* = 0.003, transient vs. sustained: *p* = 0.0156. For 140 Hz DBS, paired t-test, transient: *p* = 3.1 × 10^−4^, df = 25; sustained: *p* = 0.001, transient vs. sustained: *p* = 0.78. Comparison between 40 Hz and 140 Hz DBS, independent t-test, df = 46, transient: *p* = 0.045; sustained: *p* = 0.79. **d-f** Spike rate and corresponding quantifications. **d, e** Population-averaged spike rate during 40 Hz DBS (**d,** *n* = 22) and 140 Hz DBS (**e**, *n* = 26). Shaded gray area is SEM. **f** Violin plots of spike rate change from baseline during the transient and sustained periods of 40 Hz and 140 Hz DBS. For 40 Hz DBS, paired t-test, df = 21 neurons, transient: *p* = 0.0013; sustained: *p* = 0.002, transient vs. sustained: p = 0.19. For 140 Hz DBS, paired t-test, transient: *p* = 0.0071, df = 25; sustained: *p* = 0.11, transient vs. sustained: p = 0.01. Comparison between 40 Hz and 140 Hz DBS, independent t-test, df = 46, transient: *p* = 0.97; sustained: *p* = 0.089. **g**-**i** Time-frequency spectrum power of Vm and corresponding quantifications. Population-averaged Vm power during 40 Hz DBS (**g**, *n* = 22) and 140 Hz DBS (**h,** *n* = 26). **i** Violin plots of Vm 40 Hz or 140 Hz power change relative to the baseline during the transient and sustained periods of 40 Hz and 140 Hz DBS. For 40 Hz DBS, paired t-test, df = 21 neurons, transient: *p* = 1.05 × 10^−4^; sustained: *p* = 1.23 × 10^−4^, transient vs. sustained: *p* = 0.94. For 140 Hz DBS, paired t-test, transient: *p* = 9.77 × 10^−6^, df = 25; sustained: *p* = 0.0017, transient vs. sustained: *p* = 5.76 × 10^−5^. Comparison between 40 Hz and 140 Hz DBS, independent t-test, df = 46, transient: *p* = 4.87 × 10^−4^; sustained: *p* = 4.05 × 10^−4^. ns = non-significant, *<0.05, **<0.01, and ***<0.001. All paired or independent t-tests are two-sided. Source data are provided as a Source Data file.

We found that, at the population level, Vm depolarization closely tracked the overall time course of the DBS pulse trains (Fig. 2a–c), while we also observed variability across neurons (Supplementary Fig. 1). Interestingly, 40 Hz DBS evoked Vm depolarization ramped gradually over the first couple hundred milliseconds following the onset, whereas 140 Hz DBS evoked Vm depolarization rose to peak within tens of milliseconds. Thus, we analyzed the initial transient response within 150 ms of stimulation onset (transient period), and the sustained response during the remaining 150 ms-1 s period of each stimulation pulse train (sustained period). We found that 40 Hz DBS led to significant Vm depolarization during both periods compared to the pre-stimulation baseline (paired t-test, degree of freedom (df) = 21 neurons, transient: *p* = 0.002; sustained: *p* = 0.003). Similarly, 140 Hz DBS led to prominent Vm depolarization during both periods (paired t-test, transient: *p* = 3.1 × 10^−4^, df = 25; sustained: *p* = 0.001, df = 25). However, 140 Hz DBS induced a significantly stronger depolarization than 40 Hz DBS during the transient period, but not the sustained period (independent t-test, df = 46, transient: p = 0.045; sustained: *p* = 0.79). Following stimulation offset, Vm quickly returned to the baseline level for both DBS conditions (paired t-test, 40 Hz DBS: *p* = 0.19, df = 21; 140 Hz DBS*: p* = 0.84, df = 25).

We then examined how DBS-induced Vm depolarization influences spiking output. We found that 40 Hz DBS led to a sustained increase in firing rate throughout the stimulation period (Fig. 2d) (paired t-test compared to pre-stimulation baseline, transient: *p* = 0.0013, df = 21; sustained: *p* = 0.002, df = 21). Following stimulation offset, the spike rate quickly returned to the baseline level (paired t-test, *p* = 0.49, df = 21). Upon 140 Hz DBS, we found a strong increase in firing rate during the transient period compared to the pre-stimulation baseline (paired t-test, *p* = 0.0071, df = 25), followed by a small but non-significant increase in firing rate during the sustained period (Fig. 2e, paired t-test, *p* = 0.11, df = 25). We found no significant difference in evoked firing rate change between 40 Hz DBS and 140 Hz DBS during both the transient and the sustained periods (independent t-test, transient: *p* = 0.97, df = 46; sustained: *p* = 0.089, df = 46). Together, these results demonstrate that both 40 Hz and 140 Hz DBS powerfully depolarize Vm without suppressing spike rate. While 40 Hz DBS substantially increased neurons spike rate throughout the stimulation duration, 140 Hz DBS transiently increased spike rate within 150 ms of DBS onset.

### DBS entrains Vm at the stimulation frequency

Neurons are capable of following certain rhythmic inputs, a phenomenon known as entrainment, an important network communication mechanism^36,37^. Entrainment by DBS has been proposed as a potential therapeutic mechanism, where DBS-mediated neural activity or entrainment interferes with a neuron’s ability to process synaptic inputs leading to disruption of pathological network connectivity^19^. Thus, we next examined whether DBS could entrain or evoke precisely timed neuronal responses throughout the entire stimulation pulse duration. We computed the wavelet power spectrum of the Vm and examined changes in either 40 Hz or 140 Hz power during DBS pulse trains relative to baseline. We found that 40 Hz DBS powerfully entrained Vm, leading to a prominent 40 Hz component in the Vm power spectrum (Fig. 2g). It quickly emerged at the stimulation onset, and sustained throughout the stimulation pulse train duration (paired t-test comparing stimulation to the pre-stimulation baseline, transient: *p* = 1.05 × 10^−4^, df = 21; sustained: *p* = 1.23 × 10^−4^, df = 21, Fig. 2h). 140 Hz DBS also entrained Vm at 140 Hz (paired t-test, transient: *p* = 9.77 × 10^−6^, df = 25; sustained: *p* = 0.0017, df = 25; Fig. 2h, i), though the magnitude of 140 Hz power induced by Vm entrainment at 140 Hz was significantly weaker than the 40 Hz power induced by Vm 40 Hz entrainment during both the transient and the sustained periods (independent t-test, transient: *p* = 4.87 × 10^−4^, df = 46; sustained: *p* = 4.05 × 10^−4^, df = 46; Fig. 2f). Following stimulation offset, Vm power returned to the baseline level under both DBS conditions (paired t-test, 40 Hz DBS: *p* = 0.1, df = 21; 140 Hz DBS: *p* = 0.63, df = 25). Together, these results demonstrate that 40 Hz DBS powerfully entrained Vm, whereas 140 Hz DBS had a substantially weaker but nonetheless significant entrainment effect throughout the stimulation duration.

### Individual electrical pulse-evoked Vm and firing rate changes are DBS frequency dependent

**Fig. 3:**
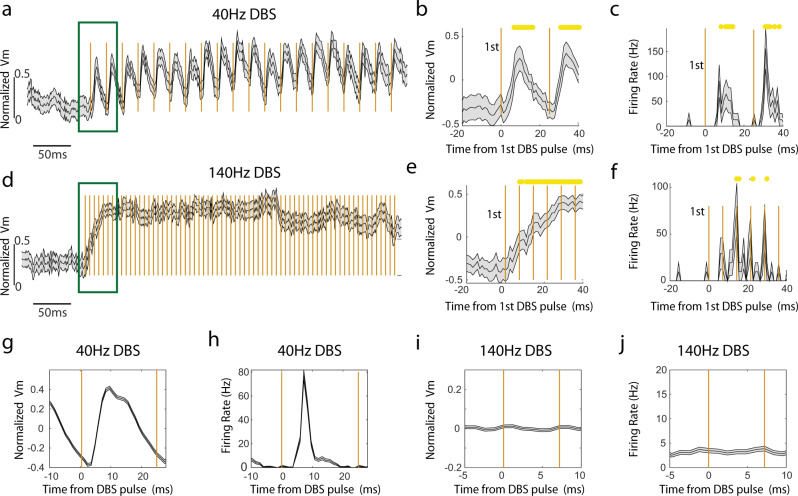
Individual electrical pulse evoked Vm and firing rate modulations are DBS frequency dependent. **a** The population-averaged Vm trace before, during and after 40 Hz DBS with the pulse onset times indicated by the golden vertical lines. Green box indicates the time windows where zoom-in versions are shown in **b, e**. Shaded area represents SEM. Vm is calculated as the fluorescence at each time point divided by the average amplitude of all spikes detected in a recording session for a given neuron. DBS-induced Vm changes were computed as normalized Vm by subtracting the mean of the Vm during the prestimulation baseline period. **b** The population-averaged Vm trace (*n* = 22 neurons) around 40 Hz DBS onset. Shaded area represents SEM. Time window as indicated in (**a**). Time zero corresponds to the first DBS pulse time. Yellow dots represent Vm modulations during DBS that are two standard deviations from the baseline Vm distribution. **c** Same as (**b**), but showing the population-averaged firing rate. **d–f** Same as (**a**-**c**), but for 140 Hz DBS (*n* = 26 neurons). **g** Population-averaged Vm aligned to the onset of all pulses during 40 Hz stimulation. Golden lines indicate the pulse onset times. Shaded area represents SEM. **h** Same as (**g**), but for firing rate. **i**-**j** Same as (**g**, **h**), but for 140 Hz DBS.

To gain insights into how DBS alters Vm and spiking at a finer temporal scale, we aligned Vm and spike rate to each individual electrical pulse within the DBS pulse trains. Following the onset of the first electrical pulse delivered at 40 Hz (Fig. 3a–c), Vm started to rise after a brief delay of a couple of milliseconds, and was significantly above the baseline by 7 ms for Vm depolarization (*p* < 0.05, paired t-tests comparing each time point relative to the pre-stimulation baseline, *n* = 22), and peaked at 14.1 ± 0.85 ms (mean ± SEM, *n* = 22). Similarly, spike rate increase became significant at 7 ms (*p* < 0.05, paired t-tests) after stimulation onset and peaked at 10.4 ± 4.7 ms (mean ± SEM). After the peak, both Vm and spike rate dropped towards the baseline. By the time when the second electrical pulse occurred at 25 ms after the first pulse, both Vm and spike rate dropped enough that the second pulse was able to evoke similar Vm depolarization and spike rate increase as the first pulse. To further quantify the change evoked by each electrical pulse, we computed the average Vm and spike rate across all pulses delivered during the entire DBS pulse trains. Similar to that observed following the first electrical pulse, across all pulses, Vm depolarization peaked at 9.2 ± 1.3 ms (mean ± SEM, *n* = 22 neurons) after the pulse onset and spike rate peaked at 13.2 ± 1.8 ms (Fig. 3g, h). Thus, with 40 Hz DBS, the membrane voltage depolarization evoked by each individual electrical pulse has enough time to repolarize towards the baseline, allowing the neurons to respond to subsequent pulses, leading to an overall entrainment of Vm and spike rate, and robust Vm depolarization and spike rate increase throughout the entire duration of the stimulation pulse trains.

When we examined the temporal dynamics of Vm and spike rate modulation following each individual pulse delivered at 140 Hz (Fig. 3d–f), we found that Vm and spike rate failed to follow the stimulation pulses even at the beginning of the pulse trains, in sharp contrast to that observed with 40 Hz DBS. Even though the first electrical pulse evoked Vm depolarization took about 8 ms to become significant (*p* < 0.05, paired t-tests comparing each time point relative to the pre-stimulation baseline, *n* = 26), similar to that seen with 40 Hz DBS, Vm continued to rise when the second pulse was delivered at 7.1 ms after the first without decreasing towards the baseline (Fig. 3d, e). As a result, DBS evoked Vm depolarization ramped over the first ~40 ms when 6–7 electrical pulses were delivered. Because of the generally weak effect of 140 Hz DBS on spike rate (Fig. 2h), we found that the increase in spike rate did not reach significance until 14 ms after the first pulse (*p* < 0.05, paired t-tests comparing each time point relative to the pre-stimulation baseline, *n* = 26), and the spike rate increase lacked a clear peak. More strikingly, when we examined the average evoked Vm and spike rate across all pulses delivered during the entire 140 Hz DBS pulse trains, we detected no clear change in Vm or spike rate following each individual pulse (Fig. 3i, j). Together, these results demonstrate that Vm and spiking of CA1 neurons can better follow individual electrical pulses during 40 Hz DBS than 140 Hz DBS.

### DBS entrains CA1 spike timing at the stimulation frequency

**Fig. 4:**
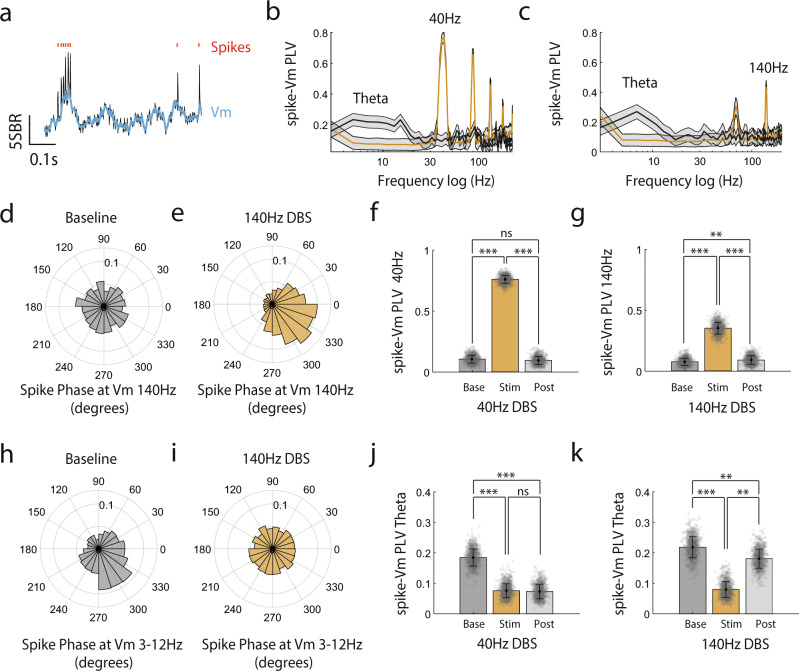
DBS entrains CA1 spiking at the stimulation frequency while reducing theta-rhythmic spike output. **a** Example SomArchon trace (black) of a CA1 neuron with Vm highlighted blue (smoothed ± 6 ms), and spike times marked with red ticks. **b** Population phase-locking value (PLV) of spikes to Vm across frequencies. Spikes from all neurons were concatenated for PLV computation (40 Hz, n = 1972, 140 Hz, *n* = 1867). Black line represents spike-Vm PLV during baseline period and golden line represents spike-Vm PLV during 40 Hz DBS period. Shaded area is standard deviation estimated through bootstrapping. Frequency-axis is on a logarithmic scale. **c** Same as (**b**), but for 140 Hz DBS. **d** The population polar histogram of spike times relative to the phase of Vm filtered at 140 Hz using a Butterworth filter during the baseline period. **e** Same as (**d**), but during the 140 Hz DBS period. **f** Quantification of spike-Vm PLV at the DBS 40 Hz entrainment frequency during baseline (Base), 40 Hz DBS (Stim) and post-stimulation (Post) periods. Black error bars are standard deviation. Individual data points represent population mean samples obtained by bootstrapping. **g** Same as (**f**), but for 140 Hz DBS. **h** The circularly averaged population polar histogram of spike times relative to the phase of Vm filtered between 3–12 Hz (theta-frequency range). **i** Same (**h**), but during the 140 Hz DBS period. **j** Quantification of spike-Vm PLV in the theta-frequency range, averaged across 3–12 Hz, during baseline (Base), 40 Hz DBS (Stim) and poststimulation period (Post). Individual data points represent population mean samples obtained by bootstrapping. **k** Same as (**j**), but for 140 Hz DBS. Statistics are based on permutation testing comparing estimated values to permutation-derived null distributions, ns = non-significant, **<0.01, and ***<0.001. Source data are provided as a Source Data file.

Spike initiation is determined by somatic Vm, which reflects a spatiotemporal transformation of synaptic inputs shaped by the intrinsic membrane ionic conductance^38,39^. Since DBS generates powerful Vm depolarization and entrains Vm to the stimulation frequency (Figs. 2g–i, 3), we further examined how DBS evoked cellular membrane voltage changes relate to spike timing by computing the spike phase locking value (PLV^40^) relative to Vm oscillation phase. PLV measures the consistency (mean vector length) of spike timing relative to a particular phase of a Vm oscillation. Due to the low spike counts in some neurons, particularly during the pre-DBS baseline and post-DBS periods, we computed population spike PLV by aggregating all spikes in all recorded neurons. We found that DBS significantly increased spike-Vm PLV (Fig. 4b–g) at the stimulation frequency, for both 40 Hz DBS (permutation test, baseline vs DBS, *p* < 0.001, DBS vs post-DBS, *p* < 0.001, baseline vs post-DBS, *p* > 0.05) and 140 Hz DBS (permutation test, baseline vs DBS, *p* < 0.001, DBS vs post-DBS, *p* < 0.001, baseline vs post-DBS, *p* < 0.01). 40 Hz DBS led to stronger spike-Vm PLV than 140 Hz DBS (permutation test, 40 Hz DBS vs 140 Hz DBS, *p* < 0.001), suggesting a stronger entrainment effect for 40 Hz DBS than 140 Hz DBS. Due to the harmonic effect of power spectrum analysis, spike-Vm PLV was also present at the harmonic frequencies for the 40 Hz DBS (80 Hz, 120 Hz and 160 Hz) and the sub-harmonic frequencies for 140 Hz DBS (70 Hz) (Fig. 4b, c).

During DBS, because of the elevated spike rates, there were enough spikes in most neurons that allowed us to compute spike-Vm PLV at the individual neuron level. Consistent with the observation at the population level, DBS led to significant entrainment of spike-Vm PLV at 40 Hz during 40 Hz DBS (paired t-test, DBS vs shuffled data, *p* = 5.77 × 10^−8^, df = 19, only neurons with >5 spikes during stimulation period, Supplementary Fig. 4) and at 140 Hz during 140 Hz DBS (paired t-test, DBS vs shuffled data, *p* = 2.1 × 10^−4^, df = 22). 40 Hz DBS led to stronger spike-Vm PLV than 140 Hz DBS across neurons (independent t-test, *p* = 6.5 × 10^−6^, df = 41, Supplementary Fig. 4), again confirming that 40 Hz DBS exhibits a more powerful spike entrainment effect than 140 Hz DBS.

### DBS reduces an individual neuron’s ability to spike in response to hippocampal network theta rhythmic activity patterns

Rhythmic coordination of circuit activity results in synchronized synaptic inputs that entrain spike timing^41–45^. In the hippocampal CA1, there are prominent and persistent theta frequency (3–12 Hz) oscillations that are crucial for hippocampal-dependent spatial memory and spatial navigation^41,45–47^, and it has been widely documented that CA1 network theta oscillations entrain spike timing. At the single-cell level, theta oscillations have also been reported in the Vm of individual CA1 neurons, which regulates the timing of spike output^30,47,48^. Consistent with prior studies, we detected significant spike-Vm PLV in the theta frequency range (Fig. 4h–k) in the baseline period (both 40 Hz and 140 Hz DBS conditions, permutation test, baseline vs shuffled, *p* < 0.01). Strikingly, DBS led to a strong and significant reduction of spike-Vm theta frequency PLV for both 40 Hz DBS (permutation test, baseline vs DBS, *p* < 0.001, DBS vs post-DBS, *p* > 0.05, baseline vs post-DBS, *p* < 0.001) and 140 Hz DBS (permutation test, baseline vs DBS, *p* < 0.001, DBS vs post-DBS, *p* < 0.01, baseline vs post-DBS, *p* < 0.01). Thus, DBS at either 40 Hz or 140 Hz can effectively suppress individual neuron’s ability to transform hippocampal network theta frequency inputs to precisely timed spike output.

### Simultaneous SomArchon imaging and optogenetic CoChR activation to quantify a neuron’s ability to respond to somatic depolarizing inputs during DBS

**Fig. 5:**
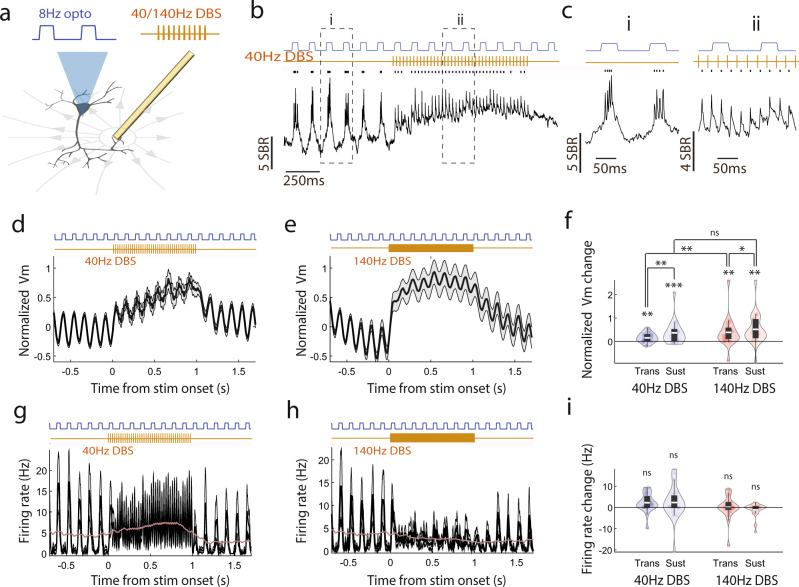
DBS evokes additional Vm depolarization without changing the spike rate in the presence of CoChR optogenetic inputs. **a** Illustration of simultaneous CoChR-evoked membrane depolarization and SomArchon voltage imaging during DBS. Optogenetic blue light stimulation was delivered wide-field over an area of 350 microns defined by the 40x objective. Schematic neuron was adapted from scridraw.io (doi.org/10.5281/zenodo.3925905). **b** An example CA1 neuron’s SomArchon fluorescence trace (black) and spikes (black ticks) during 8 Hz CoChR activation (blue line) and 40 Hz DBS (gold line). 8 Hz CoChR activation occurred throughout the 3-second trial, whereas 40 Hz DBS occurred for 1 second in the middle of each trial. **c** Zoom-in view of the periods indicated by the dashed lines in (b), during the baseline (i) and the DBS period (ii). **d** Population-averaged Vm during simultaneous 8 Hz optogenetic activation (blue) and 40 Hz DBS (gold). Vm is calculated as the fluorescence at each time point divided by the average amplitude of all spikes detected in a recording session for a given neuron (*n* = 20). DBS-induced Vm changes were computed as normalized Vm by subtracting the mean of the Vm during the pre-stimulation baseline period. Shaded area represents SEM. **e** Same as (**d**), but with 140 Hz DBS (*n* = 21). **f** Quantification of the transient (0–0.15 sec) and the sustained (0.15–1 sec) Vm depolarization induced by either 40 Hz or 140 Hz DBS, in the presence of optogenetic activation. Data are visualized as violin plots with the outer shape representing the data kernel density and a box plot showing the interquartile range (1x, 1.5x). The white lines in the boxes are the mean. For 40 Hz DBS, paired t-test, df = 19, transient: *p* = 0.0036; sustained: *p* = 0.004, transient vs. sustained: *p* = 0.028. For 140 Hz DBS, paired t-test, df = 20; transient: *p* = 0.01, sustained: *p* = 0.0025, transient vs. sustained: *p* = 0.0187. Comparison between 40 Hz and 140 Hz DBS, independent t-test, df = 39, transient: *p* = 0.0082; sustained: *p* = 0.42. (**g**). Population-averaged firing rate during simultaneous 8 Hz optogenetic activation (blue) and 40 Hz DBS (brown, *n* = 16 neurons). Shaded area represents SEM. The purple line represents the further smoothed firing rate (300 ms rectangular smoothing). **h** Same as (**g**), but for population-averaged firing rate with 140 Hz DBS (*n* = 17). **i** Violin plots of the transient (0–0.15 sec) and the sustained (0.15–1 sec) firing rate changes relative to the baseline induced by either 40 Hz or 140 Hz DBS, in the presence of optogenetic activation. For 40 Hz DBS, paired t-test, df = 19, transient: *p* = 0.062; sustained: *p* = 0.28. For 140 Hz DBS, paired t-test, df = 20; transient: *p* = 0.76, sustained: *p* = 0.087. Given DBS conditions were not significant from baseline, we did no comparison across DBS conditions. Two-sided paired t-test for within DBS condition statistics and two-sided independent t-test for between DBS condition statistics. ns, non-significant, *<0.05, **<0.01, and ***<0.001. Source data are provided as a Source Data file.

Theta rhythmic spiking has been shown to be coordinated across broad hippocampal and cortical brain structures^45,49,50^, which produces rhythmic synaptic inputs to CA1 that contribute to the pacing of CA1 neuronsʼ Vm and spiking. Thus, DBS-induced changes in spike-Vm theta phase locking could be through disrupting theta rhythmic synaptic inputs originating from interconnected brain structures or through affecting the somatic Vm of the recorded neurons directly. To further explore DBS effect on neurons’ ability to respond to somatic inputs, we expressed the blue-light activated and soma-targeted cation channel CoChR in the same neurons that also expressed SomArchon (Fig. 5a). Blue-light wide-field illumination of CoChR would thus directly depolarize the soma of the recorded neuron, which allowed us to determine how DBS influences individual neuron’s response to somatic transmembrane voltage depolarization inputs mediated by CoChR optogenetic activation. Specifically, we illuminated the imaging field (about 350 microns in diameter) with pulsed blue light (470 nm LED) at 8 Hz (theta frequency) to optogenetically depolarize CoChR-expressing neurons. Because SomArchon is insensitive to the blue light used for CoChR activation^30,51^, we were able to perform simultaneous SomArchon voltage imaging and CoChR activation in the same neuron while delivering DBS. For each 3-second-long trial, neurons were optogenetically stimulated throughout the entire trial period, and 40 Hz or 140 Hz DBS was delivered for 1 second in the middle (Fig. 5a).

We found that blue light induced CoChR activation led to powerful membrane depolarization in individual CA1 neurons, and each light pulse evoked precisely timed spiking during the baseline period before DBS (Fig. 5b, c, Supplementary Fig. 5). Both Vm and spikes followed rhythmic 8 Hz CoChR activation, consistent with the general observation that CA1 neurons are easily entrained by theta frequency inputs. During DBS, at 40 Hz and 140 Hz, we detected additional Vm depolarization on top of CoChR-evoked Vm depolarization (Fig. 5d–f). However, we failed to detect any additional spike rate increase during DBS in the presence of CoChR activation, compared to before DBS or after DBS (Fig. 5g–i). It is possible that the neuronal firing rate has already peaked with CoChR activation, and the additional Vm depolarization generated by DBS is insufficient to further increase the spike rate. Thus, DBS-induced spike rate change depends on the membrane voltage or the excitability state of the neuron.

**Fig. 6:**
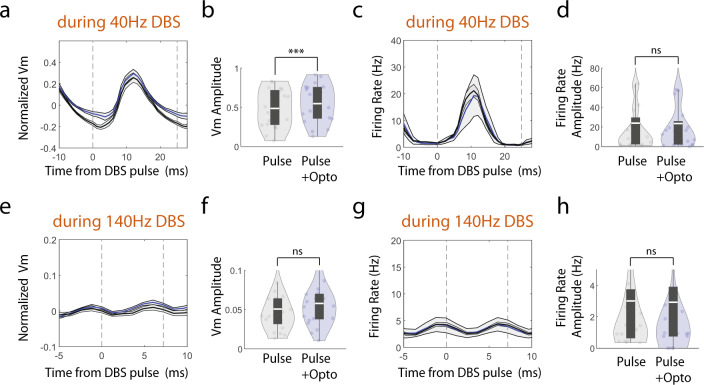
Individual electrical pulse evoked Vm and firing rate changes are differentially modulated by optogenetic depolarization. **a** Population-averaged Vm aligned to individual electrical pulses during 40 Hz DBS with simultaneous optogenetic CoChR activation (blue line) and without CoChR activation (black line). Dashed lines indicate the onset of individual DBS electrical pulse times. Pulse-averaged Vm was first computed for each neuron and then averaged across neurons. Normalized Vm was then computed by subtracting the mean of the population pulse averaged Vm during the 10 ms (40 Hz DBS) or 5 ms (140 Hz DBS) time period before pulse onset. Shaded area represents SEM. **b** Electrical pulse evoked Vm change, computed as the difference between the maximum minus the minimum of the pulse averaged Vm per neuron. Data are visualized as violin plots with the outer shape representing the data kernel density and a box plot showing interquartile range (1x, 1.5x). The white lines in the boxes are the mean. Paired t-test, p = 3.3 × 10^−5^, df = 19. **c, d** Same as (**a,b**), but for population-averaged firing rate aligned to individual electrical pulses during 40 Hz DBS. Paired t-test, p = 0.7, df = 19 (**e-h**) Same as (**a-d**), but for 140 Hz DBS. Paired t-test (Vm), p = 0.088, df = 20. Paired t-test (Firing rate), p = 0.15, df = 20. Two-sided paired t-test for all tests. ns = non-significant and ***<0.001. Source data are provided as a Source Data file.

To further evaluate the membrane voltage-dependent effect of DBS on Vm and spike rate, we examined individual electrical pulse-evoked responses with versus without optogenetic CoChR activation. Since CoChR activation was achieved by illuminating neurons with 42 ms-long blue light pulses every 125 ms (8 Hz optogenetic), we compared the evoked responses by DBS pulses that occurred during blue light illumination versus without blue light. We found that individual pulses during 40 Hz DBS produced smaller Vm depolarization with simultaneous CoChR activation than without CoChR (Fig. 6a, b, paired t-test, *p* = 3.3 × 10^−5^, df = 19), consistent with a ceiling effect of Vm depolarization where action potential generation limits the magnitude of the subthreshold Vm changes. This is further supported by the observation that DBS-evoked spike rate increase remained the same with versus without CoChR activation (Fig. 6c, d, paired t-test, *p* = 0.7, df = 19). Since individual electrical pulses during 140 Hz DBS produced little Vm modulation without CoChR activation (Fig. 3i), it is not surprising that Vm and spike rate modulation by individual pulses of 140 Hz DBS was not altered by CoChR activation (Fig. 6e–h, paired t-test, Vm, *p* = 0.088, df = 20, firing rate, *p* = 0.15, df = 20). Since CoChR is a light-activated cation channel, CoChR activation depolarizes membrane voltage and increases membrane conductance. While we cannot separate the effect of membrane depolarization versus conductance increase during CoChR activation, the observed difference in DBS-mediated Vm depolarization with versus without CoChR activation during 40 Hz, but not 140 Hz DBS, demonstrates that DBS-evoked membrane depolarization relies on the biophysical properties of the stimulated neuron.

### DBS reduces a neuron’s ability to respond to somatic depolarization inputs evoked by optogenetic activation

**Fig. 7:**
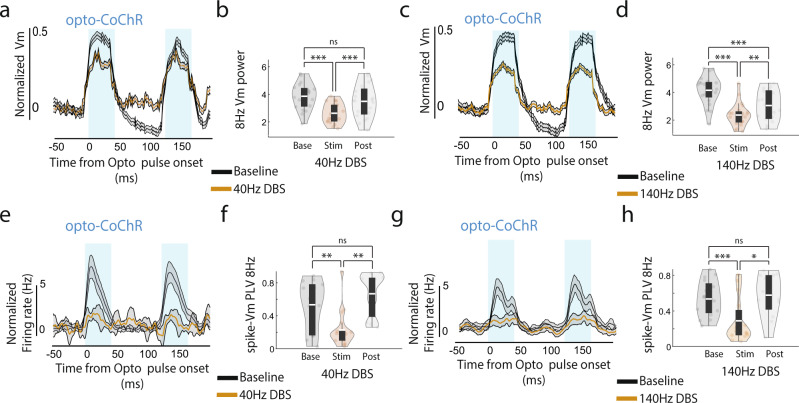
DBS reduces a neuron’s ability to respond to somatic membrane depolarization inputs evoked optogenetically. **a** Population-averaged Vm aligned to blue-light CoChR pulse onsets during baseline (back line) and 40 Hz DBS (golden line). Shaded area represents SEM. The vertical blue shaded area corresponds to the optogenetic CoChR activation period (42 ms). The average Vm during the baseline period (−50 ms to 0 ms) was subtracted from the Vm. **b** Quantification of 8 Hz CoChR-evoked Vm power change during baseline (Base), 40 Hz DBS (Stim) and post-stimulation period (Post). Data are visualized as violin plots with the outer shape representing the data kernel density and a box plot showing interquartile range (1x, 1.5x). The white lines in the boxes are the mean. Stim vs. Baseline, paired t-test, *p* = 5.22 × 10^−7^, df = 19. Stim vs. Post, paired t-test, *p* = 5.6 × 10^−4^, df = 19, Post vs. Baseline, paired t-test, *p* = 0.07, df = 19. **c**, **d** Same as (**a-b**), but for 140 Hz DBS condition. Stim vs. Baseline, paired t-test, *p* = 5.66 × 10^−7^, df = 20. Stim vs. Post, paired t-test, *p* = 0.0024, df = 20, Post vs. Baseline,, paired t-test, *p* = 6.22 × 10^−4^, df = 20. **e** Same as (**a**), but for firing rate. Firing rate was normalized by subtracting the average firing rate during the baseline period (−50 ms to 0 ms). **f** Violin plots of spike phase-locking value (PLV) to 8 Hz Vm during baseline (Base), 40 Hz DBS period (Stim) and post-stimulation period (Post). Only neurons included with >5spikes. Stim vs. Baseline, paired t-test, *p* = 0.008, df = 14. Stim vs. Post, paired t-test, *p* = 0.0065, df = 9, Post vs. Baseline, paired t-test, p = 0.49, df = 8. **g**, **h** Same as (**e**, **f**), but for 140 Hz DBS. Stim vs. Baseline, paired t-test, *p* = 0.0008, df = 11. Stim vs. Post, paired t-test, *p* = 0.0127, df = 9, Post vs. Baseline, paired t-test, p = 0.82, df = 10. Two-sided paired t-test for all tests. ns, non-significant, *<0.05, **<0.01, and ***<0.001. Source data are provided as a Source Data file.

To further assess DBS effect on individual neuronsʼ ability to respond to inputs, we analyzed the reliability of CA1 neurons in following 8 Hz optogenetic somatic stimulation. While somatic CoChR stimulation does not model dendritic synaptic inputs, it approximates somatic voltage dynamics after integrating dendritic inputs. Thus simultaneous optogenetic somatic depolarization and DBS allows us to experimentally test how DBS influences individual neuronsʼ ability to follow depolarizing inputs that reflect network inputs. Since Vm was reliably paced by 8 Hz optogenetic activation of CoChR, we first computed 8 Hz Vm power before, during, and after DBS (Fig. 7a, b). With 40 Hz DBS, we found a significant reduction of 8 Hz Vm power during DBS compared to the pre-stimulation baseline (paired t-test, p = 5.22 × 10^−7^, df = 19), which largely recovered to the baseline level after DBS (paired t-test, *p* = 0.07, df = 19). With 140 Hz DBS (Fig. 7c, d), 8 Hz Vm power decreased not only during DBS (paired t-test, p = 5.66 × 10^−7^, df = 20), but remained suppressed after DBS compared to the baseline (paired t-test, p = 6.22 × 10^−4^, df = 20). The reduction of Vm 8 Hz power during 140 Hz DBS was significantly greater than during 40 Hz DBS (independent t-test, *p* = 0.046, df = 39), but not after DBS (independent t-test, p = 0.055, df = 39). Thus, DBS reduced Vm responding to rhythmic 8 Hz inputs that are otherwise powerful at entraining CA1 neurons. Consistent with a loss of Vm entrainment to optogenetically induced 8 Hz membrane depolarization, we found that during both 40 Hz and 140 Hz DBS, CoChR largely failed to evoke precisely timed spikes (Fig. 7e, g). Spike-Vm PLV significantly reduced during DBS compared to the pre-stimulation baseline (Fig. 7f, h, only neurons with >5spikes during stimulation and baseline period included, paired t-test, 40 Hz DBS: *p* = 0.008, df = 14; 140 Hz DBS: *p* = 0.0008, df = 11), which quickly recovered after DBS to the baseline level for both DBS conditions (paired t-test, >5spikes during baseline and post-stimulation period included, 40 Hz DBS: *p* = 0.49, df = 8; 140 Hz DBS: *p* = 0.82, df = 10).

**Fig. 8:**
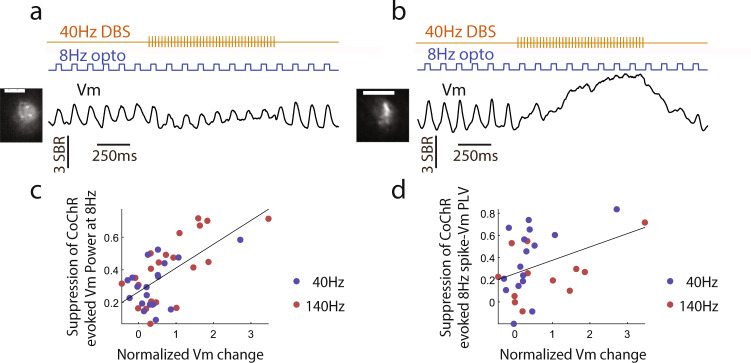
DBS-induced membrane depolarization predicts suppression of optogenetic inputs. **a** A single neuron example showing the trial-averaged Vm during 8 Hz CoChR activation and 40 Hz DBS. The Vm has been smoothed with a 30 ms rectangular window to better highlight the depolarization effect in response to 8 Hz CoChR activation. This neuron exhibited prominent optogenetics-evoked Vm depolarization without DBS, and reduced Vm depolarization during 40 Hz DBS. Average SomArchon fluorescence of the example neuron is shown in the left image. Scale bar, 15 µm (**b**) Same as (**a**), but another neuron example with strong Vm depolarization during 40 Hz DBS. **c** The reduction of optogenetically induced 8 Hz Vm power is shown as a function of Vm change (DBS – Baseline) during 40 Hz (blue dots) and 140 Hz (red dots) DBS. Vm change is normalized by the average spike amplitude for each recorded neuron. Each dot represents a neuron (*n* = 41 neurons total). Neurons with stronger DBS-evoked Vm depolarization exhibit greater suppression of the 8 Hz optogenetically induced Vm power. The black line represents the fitted linear regression line. **d** The reduction of optogenetically induced 8 Hz spike-Vm phase locking value (PLV) is shown as a function of Vm depolarization amplitude during 40 Hz (blue dots) and 140 Hz (red dots) DBS (*n* = 28 total neurons). Neurons with less than 5 spikes during stimulation and baseline were excluded from this analysis. Source data are provided as a Source Data file.

Finally, to understand how the loss of neuronal responding to inputs relates to DBS-induced membrane depolarization, we compared the DBS effect on entrainment versus Vm depolarization. Across neurons analyzed, DBS-induced Vm depolarization is correlated with the DBS-mediated suppression of Vm entrainment by optogenetic inputs (Fig. 8, linear regression slope, r^2^ = 0.43, *p* < 1 × 10^−20^). Similarly, DBS-induced Vm depolarization is also correlated with DBS-mediated reduction of spike entrainment by optogenetic inputs (linear regression slope, r^2^ = 0.157, *p* = 0.037, only neurons with >5 spikes during stimulation and baseline). These results confirmed that DBS-induced membrane depolarization is associated with suppressed responding of individual neurons to inputs.

## Discussion

We demonstrate that hippocampal DBS powerfully depolarizes individual neuronsʼ membrane voltage, with higher-frequency 140 Hz DBS producing a more pronounced Vm depolarization than lower-frequency 40 Hz DBS. Further, DBS increased spike rates and entrained spike timing throughout the stimulation period, particularly during 40 Hz DBS. Thus, our results do not support an overall neural silencing hypothesis, as we found continued spiking throughout the stimulation period. However, DBS interferes with individual CA1 neuronʼs ability to produce precisely timed spiking output in response to hippocampal network theta rhythmicity suggesting reduced responses to synaptic inputs. Moreover, DBS at both 40 Hz and 140 Hz suppressed the temporally precise response of spike and Vm to somatic optogenetic depolarization inputs, and the magnitude of this suppression is correlated with the strength of DBS-induced Vm depolarization. Together, these results demonstrate that hippocampal DBS attenuates individual neuronsʼ responses to inputs while depolarizing Vm and entraining spike output. Thus, DBS interferes with a neuron’s ability to transform membrane voltage inputs to spiking output at the targeted area through membrane depolarization, consistent with the informational lesion or functional deafferentation hypothesis^19^. The substantial information lesion created by DBS at the individual neuron level provides a cellular mechanism to promote the suppression of pathological network synchronization and thus contributes to the network mechanisms of therapeutic DBS.

DBS has been approved to deliver chronic stimulation in the thalamus and the basal ganglia for treating the motor symptoms of Parkinson’s disease and essential tremor^1–4^, and the limbic system for reducing seizure frequencies in drug-resistant temporal epilepsy^5–7^. Additionally, close-loop DBS, also known as responsive neurostimulation, transiently delivers high-frequency DBS to seizure onset zones after detecting pathological field potential features and was recently shown in long-term studies to be also effective at reducing seizure frequencies^24,25^. Because the clinical benefit of DBS is consistent with that achieved via surgical removal or pharmacological inhibition of the targeted brain tissue, it was first thought that DBS silences neural activity. However, DBS (i.e. in the STN and Gpi) does not necessarily reduce neuronal spiking rates^1–4,18,52^. We did not detect a significant change in the overall neuronal firing rate during the 1-second long 40 Hz or 140 Hz DBS period without (Fig. 2) or with (Fig. 5) optogenetics. Thus, our single-neuron results do not support the neural silencing hypothesis. Instead, our findings demonstrated that CA1 neurons maintained their spiking activity during DBS, or slightly increased their spiking, which allows the activated neurons to engage downstream areas (i.e. subiculum) to recruit distally interconnected circuits as observed previously^52^.

The informational lesion hypothesis emphasizes the network effect of DBS^19^, where DBS suppresses the neuronal ability to transform network inputs to spiking outputs rather than simply silencing spiking activity. Our observation in the hippocampus of awake mice supports the informational lesion effect of DBS and is in line with the prior in vitro thalamic DBS study^53^. Similar neural mechanisms remain to be tested in other brain regions, i.e. the basal ganglia and the thalamus that are widely used as DBS targets.

The observed DBS-induced changes on the subthreshold membrane voltage at the soma of individual CA1 neurons and their spiking output could arise from a direct membrane response to electrical stimulation pulses, or indirect stimulation-induced synaptic inputs via orthodromic axonal spikes. CA1 neurons receive prominent long-range axonal inputs, e.g. via the Schaffer collateral pathway. Interestingly, we observed a delay of a couple of milliseconds between the onset of individual electrical pulses and the rise of Vm depolarization, which cannot be easily explained by direct membrane response to electrical pulses. Since axons are more excitable than dendrites or the cell body^22,54^, DBS-evoked subthreshold depolarization at the axon is expected to arrive at the soma on a sub-millisecond timescale due to the short chronaxie^15,54,55^. Furthermore, the observation that spikes did not occur until ~7–8 ms after the onset of individual electrical pulses of 40 Hz DBS further argues against direct antidromic activation of the stimulated neuron, since antidromic spiking typically has a latency shorter than a couple of milliseconds given the axon conduction velocity^56,57^. Finally, individual pulses delivered at 140 Hz failed to evoke prominent changes in Vm or spike rate, consistent with the delayed response to DBS. Together, the delay in the evoked Vm depolarization and spiking after individual pulses delivered at 40 Hz, and the lack of effects on Vm and spiking after individual pulses delivered at 140 Hz DBS, are suggestive of the synaptic mediated DBS effects shown by previous in vitro thalamic intracellular study^53^, and the altered evoked field potentials in patient Gpi during prolonged DBS^18^.

It is possible that chronic DBS recruit additional intrinsic membrane ionic conductances that contribute to the observed delay in DBS evoked Vm and spiking, though this cannot account for the observed delayed response after the very first pulse within a DBS train. For example, the membrane depolarization evoked by an individual electrical pulse may be counterbalanced by longer-lasting hyperpolarizing currents activated by previous electrical pulses during chronic DBS, where depolarization gradually overcomes hyperpolarization to generate delayed activation after each pulse. While we cannot determine whether DBS recruits slow voltage-gated hyperpolarization currents (i.e. m-current), our observation that electrical pulses delivered during optogenetic CoChR excitation did not lead to hyperpolarization argues against a direct activation of inhibitory synapses by DBS. Thus, even though our results point to a synaptic effect of DBS, we cannot rule out the recruitment of intrinsic ionic currents during sustained DBS. Despite our results indicate a synaptic mechanism underlying the DBS-induced membrane voltage effects, the direct contribution of the electrical field to somatic membrane potential changes remains unclear. Future experiments are needed to directly test whether the observed DBS-induced membrane potential effects in CA1 neurons are due to synaptic transmission. Further, future studies applying DBS with varying stimulus amplitude and pulse width, in addition to stimulation frequency as explored in this study, will be helpful in teasing apart the contribution of synaptic mechanisms from direct ion channel effects.

Over the entire 1-second-long period when trains of DBS pulses were delivered, we observed striking temporal differences in Vm depolarization modulations. 140 Hz DBS led to a rapid increase in Vm depolarization that peaked within ~40 ms and the depolarization lasted throughout the stimulation period. In contrast, 40 Hz DBS evoked gradual Vm depolarization that took over a couple hundred milliseconds to reach the plateau. Given our observation of the delay in individual pulse-induced depolarization, a possible explanation for the difference observed in such Vm depolarization kinetics is the temporal summation of post-synaptic responses. We observed DBS pulse induced Vm depolarization followed by repolarization over the 25 ms period, the inter-pulse interval during 40 Hz DBS (see Fig. 3b in 40 Hz DBS), which is consistent with the time scales of postsynaptic potentials (i.e. AMPA). It is well-known that high-frequency stimulation leads to temporal summation of synaptic EPSPs, which likely underlies the temporal differences in the summation of Vm depolarization evoked by trains of electrical pulses delivered at 40 Hz versus 140 Hz. Future work is needed to directly identify which synaptic mechanisms, excitatory or inhibitory, underlie the DBS-induced Vm depolarization in the hippocampus in the awake brain.

The temporal resolution of our SomArchon voltage imaging was limited by the sampling rate of the sCMOS camera (828 Hz), which unfortunately was not sufficient to detect the sub-millisecond DBS biphasic pulse waveforms. Additionally, the rise and decay time constants of the voltage indicator SomArchon used here, though fast enough to capture individual spikes (rising/decay constants = 0.61/1.1 ms respective^30^), are nonetheless not sufficient to detect the sub-millisecond DBS pulse waveforms. Continued improvement of ultrafast voltage imaging capabilities, such as multi-kHz voltage imaging^58^, will answer whether cellular membrane voltage can follow the waveforms of individual DBS pulses.

Hippocampal DBS is effective at reducing seizure frequencies in drug-resistant temporal lobe epilepsy patients^5–7,33,59,60^. We observed that DBS suppresses the ability of individual neuronsʼ somatic voltage to follow patterned membrane depolarization evoked by optogenetic activation of CoChR, suggesting that DBS could disrupt neuronal responses to pathological synaptic input patterns associated with seizure risks. Additionally, the strength of DBS-induced suppression of neuronal ability to process inputs is correlated with the magnitude of the evoked membrane depolarization. These findings are consistent with the in vitro observation that strong DBS-induced membrane depolarization suppressed epileptic activity in hippocampal slices^61^. Even though both low and high-frequency DBS effectively suppresses neuronsʼ ability to follow input patterns, high-frequency DBS does not produce strong spike or Vm entrainment to DBS pulse frequencies, which could be beneficial in avoiding DBS-induced over-synchronization of the targeted neural circuits. In contrast, the prominent spike entrainment effect of low-frequency DBS could be detrimental by evoking abnormal network synchronization that renders DBS ineffective or produces undesired side-effects. However, the prominent entrainment properties of lower-frequency DBS may be useful in some conditions, such as the reported memory enhancement effect during 40–50 Hz hippocampal DBS^62^. Temporal lobe epilepsy is also treated by stimulating the anterior nucleus of thalamus (ANT), part of the limbic Papez circuit, with open-loop DBS^7,32^. It will be important to study how ANT-DBS affects hippocampal circuits in comparison to hippocampal DBS. Future voltage imaging studies in epileptic animal models, in particular with DBS delivered to the common target of anterior nucleus of the thalamus^7,32^, will help produce direct experimental results on revealing the therapeutic mechanisms of DBS for seizure reduction.

In this study, we delivered 1-second-long DBS stimulation to CA1 to characterize the membrane voltage effect of DBS on individual neurons. Some clinical DBS is applied chronically, and the therapeutic outcomes and time courses are heterogeneous across disease conditions^15^. While DBS can improve certain symptoms within seconds (i.e. termination of seizure or tremor), chronic DBS over minutes to days is often needed to relieve other symptoms (i.e. bradykinesia or freezing of gait). Even though the immediate informational lesion effect of DBS observed here is relevant to the fast-acting effects of DBS therapeutics, additional plasticity mechanisms are likely involved in supporting the longer-term therapeutic effects of DBS^15^. Additionally, the immediate DBS effect observed here is also relevant to pulsed DBS in closed-loop DBS stimulation protocols^1,63–65^, where short bursts (0.2–1 sec) of high-frequency DBS (100–175 Hz) have been shown to provide longer-lasting therapeutic effects in Parkinson animal models^66^. Finally, since adult neurogenesis occurs in the dentate gyrus of the hippocampus, long-term hippocampal DBS was also shown to increase adult neurogenesis and improve cognitive memory performance^67,68^. Future studies directly assessing the plasticity effect of DBS using longer-duration stimulation protocols will help clarify how the immediate cellular effects of DBS observed here relate to their influence on longer-term plasticity changes. Different neurons with distinct biophysical properties will likely exhibit differential entrainment effects to DBS of various frequencies. For example, it has been shown that the entrainment properties of pyramidal neurons and fast spiking interneurons are different^69^. Some neurons in the STN, an important DBS target for Parkinson’s disease, have naturally high firing rates, and thus may be entrained by DBS delivered at high frequencies of >130 Hz^52^. However, at higher frequencies, such as >130 Hz, as typically used in the clinic, the somatic membrane ionic conductance time constants of many neurons likely cannot support strong sustained entrainment^52^. While both 40 Hz and 140 Hz DBS led to the suppression of neuronal responses to optogenetic inputs (informational lesion), the entrainment properties were strikingly different. Future voltage imaging analysis of electrical stimulation effects in individual neurons across a wide range of brain regions will provide a better understanding of how DBS affects neuronal responses across neural circuits, and similar analyses in pathological animal models will provide invaluable insights on the optimization of therapeutic DBS. In addition to pulse frequencies, DBS pulse amplitude is an important parameter, as reflected in clinical practice, which in this study was not systematically investigated. Nonetheless, over the DBS amplitude range tested here, we did not detect any relationship between DBS current amplitude and Vm depolarization. Future studies quantifying the effect of DBS pulse amplitude on neuronal responses, using similar approaches as highlighted in this study would provide valuable insights into the current amplitude dependence of DBS effects. Finally, future simultaneous voltage imaging analysis of multiple neurons could directly elucidate the network consequences of the DBS-created information blockade effect observed here.

## Methods

### Animal preparation

All animal experiments were performed in accordance with the National Institute of Health Guide for Laboratory Animals and approved by the Boston University Institutional Animal Care and Use and Biosafety Committees. 7 female C57BL/6 mice (Charles River Laboratories, Inc.), 8–16 weeks at the start of the study, were used for all experiments. No statistical method was used to predetermine the sample size. Mouse preparation was as described previously^30,70,71^. Custom recording apparatus consists of an imaging window coupled with a guide cannula (26 G, PlasticsOne Inc., C135GS-4/SPC) and a stainless-steel electrode for electrical stimulation (Diameter: 127 µm, PlasticsOne Inc., 005SW-30S, 7N003736501F), using super glue (Henkel Corp., Loctite 414 and Loctite 713). The imaging window consists of a stainless-steel cannula (OD: 3.17 mm, ID: 2.36 mm, 1.75 mm height, AmazonSupply, B004TUE45E), with a circular coverslip (#0, OD: 3 mm, Deckgläser Cover Glasses, Warner Instruments Inc., 64-0726 (CS-3R-0)) adhered to the bottom using a UV curable glue (Norland Products Inc., Norland Optical Adhesive 60, P/N 6001). The electrode tip protruded from the bottom of the imaging window by about 200 µm, whereas the infusion cannula was leveled with the base of the imaging window.

The recording apparatus was surgically implanted under 1–3% isoflurane anesthesia, with sustained buprenorphine administered preoperatively to provide continued analgesia for 72 hours (buprenorphine hydrochloride, 0.03 mg/kg, i.m.; Reckitt Benckiser Healthcare). A craniotomy of ~3 mm in diameter was made over the right dorsal CA1 (AP: −2 mm, ML: + 1.8 mm). A small notch was made on the posterior edge of the craniotomy to accommodate the infusion cannula and the stimulation electrode. The overlying cortex was gently aspirated using the corpus callosum as a landmark, and the corpus callosum was carefully thinned to better expose the dorsal CA1. The imaging window was then positioned in the craniotomy, and Kwik-sil adhesive (World Precision Instruments LLC, KWIK-SIL) was applied around the edges of the imaging window to hold it in place. A small ground pin was inserted into the posterior part of the brain through the skull near the lambda suture, which was used as the ground for electrical stimulation. Three small screws (J.I. Morris Co., F000CE094) were screwed into the skull, and dental cement was then gently applied to affix the imaging window, the ground pin, and an aluminum headbar posterior to the imaging window. See Fig. 1a for a diagram of the recording apparatus placement.

A week after the surgery, AAV virus was infused via an infusion cannula (33 G, PlasticsOne Inc., C315IS-4/SPC) connected to a microinfusion pump (World Precision Instruments LLC, UltraMicroPump3–4), through the previously implanted larger guide cannula. The AAV-containing infusion cannula terminated about 200 µm below the imaging window. 1000nL of either AAV9-Syn-SomArchon-BFP-p2A-CoChR (titer: 1.53e^13^ genome copies (GC)/ml, Vigene Biosciences, Inc) or AAV9-Syn-SomArchon-GFP-p2A-CoChR (titer: 5.9e^12^ GC/ml, UNC vector core) was infused at a rate of 100 nL/min, and the infusion cannula was left in place for another 10 minutes at the end of the infusion to facilitate AAV spread.

### Electrical stimulation

Electrical stimulation was delivered through an isolated pulse stimulator (Model 2100, A-M SYSTEMS). Stimulation consisted of 400 µs bipolar pulses (negative phase = 200 µs, positive phase = 200 µs). The A-M SYSTEMS pulse stimulator generates square-wave biphasic current pulses. We used either 40 Hz or 140 Hz pulse frequency. The peak amplitude per pulse ranged from 10–60 µA (corresponding to 10–64 µC/cm^2^ charge density per stimulation phase^34^) with a mean peak current of 38.3 µA and standard deviation of 11.4 µA across 7 mice. Stimulation pulse waveforms were measured with an oscilloscope (TDS2022B, Tektronix). Electrical stimulation sequences were externally triggered by TTL pulses generated by MATLAB (Mathworks Inc.) through a NI DAQ board (USB-6259, National instruments). TTL pulses were recorded at 10 kHz sampling rate using the Open Ephys platform (http://open-ephys.org).

### SomArchon voltage imaging

Habituated mice were head-fixed on an air-pressured spherical Styrofoam ball and free to run. Animals were recorded 3–4 weeks after surgery. SomArchon imaging was acquired via a customized widefield fluorescence microscope equipped with a Hamamatsu ORCA Fusion Digital sCMOS camera (Hamamatsu Photonics K.K., C14440-20UP) and a 40x NA = 0.8 water immersion objective (Nikon, CFI APO NIR). A 140 mW fiber-coupled 637 nm laser (Coherent Obis 637-140X) was coupled to a reverse 2x beam expander (ThorLabs Inc., GBE02-E) to obtain an illumination area of ~30–40 µm in diameter to minimize background fluorescence. A mechanical shutter (Newport corp., model 76995) was positioned in the laser path to control the timing of illumination via a NI DAQ board (USB-6259, National instruments). The laser beam was coupled through a 620/60 nm excitation filter (Chroma technology corp.) and a 650 nm dichroic mirror (Chroma technology corp.), and SomArchon near-infrared emission was filtered with a 706/95 nm filter (Chroma technology corp.). The fluorescence of GFP or BFP fused to SomArchon was used to localize SomArchon-expressing cells during each recording. GFP was visualized with a 470 nm LED (ThorLabs Inc., M470L3), an 470/25 nm excitation filter, a 495 nm dichroic mirror and a 525/50 nm emission filter. BFP was visualized with a 395 nm LED (ThorLabs Inc., M395L4), a 390/18 nm excitation filter, a 416 nm dichroic mirror and a 460/60 nm emission filter. SomArchon fluorescence was acquired at ~828 Hz (16 bits, 2 × 2 binning) using HCImage Live (Hamamatsu Photonics). HC Image Live data were stored as DCAM image files (DCIMG) and analyzed offline with MATLAB (Mathworks Inc.).

### Optogenetics

To excite CoChR, we used a blue 470 nm LED (ThorLabs Inc., M470L3) coupled to the widefield imaging setup with a 40x objective. The 470 nm LED was controlled by a T-Cube LED driver (ThorLabs Inc., LEDD18, low gain) that was controlled by MATLAB (Mathworks Inc.) via NI DAQ board (USB-6259, National instruments). A neutral density filter (ThorLabs Inc., ND13A, optical density 1.3) was used to reduce the LED illumination density. We used a LED light intensity of 0.01 mW/mm^2^. LED light was pulsed at 8 Hz (pulse width = 42 ms) over the imaging field of 350 μm in diameter.

### SomArchon fluorescence images pre-processing and neuron identification

All offline analyses were performed with MATLAB (2019b&2020a, Mathworks Inc.). SomArchon fluorescence images were first motion-corrected using a pairwise rigid motion correction algorithm as described previously^72^. In short, the displacement of each image was computed by identifying the max cross-correlation coefficient between each image and the reference image. Our recordings consisted of multiple multi-second trials. We therefore concatenated all trials, and then we applied the motion correction algorithm to the entire recordings. The motion-corrected image data were then used for subsequent manual neuron identification using the drawPolygon function (MATLAB). SomArchon fluorescence trace was extracted by averaging all the pixels within the identified neuron, and then detrended to correct for photobleaching using the function detrend (MATLAB). For detrending, we only considered time points before and after DBS, and excluded the DBS period due to the large subthreshold modulations induced by electrical stimulation. The DBS period was interpolated by averaging the 10 frames before and after the stimulation period making the detrending slope sensitive only to the baseline and post-stimulation fluorescence values.

### Spike identification and spike signal-to-baseline ratio (SBR) calculation

Spike detection was performed similarly as that described previously in Xiao et al.^35^. To identify spikes, SomArchon traces were first high-pass filtered (>120 Hz), and then spikes were detected as having fluorescence deflections greater than 4 standard deviations of the baseline fluctuations of the SomArchon traces. To estimate slow baseline fluctuations, we first computed a “smoothed trace” by averaging the SomArchon trace using a moving window of ±100 frames. We then removed potential spike contributions from the SomArchon trace by replacing the fluorescence values above the “smoothed trace” with the corresponding values of the “smoothed trace”. Slow baseline fluctuations were then computed as the standard deviation of the “smoothed trace”.

To estimate spike SBR, we first generated the “spike-removed SomArchon trace” by removing three data points centered at the peak of each detected spike from the SomArchon trace, and interpolated the missing data points with the surrounding data points. “The subthreshold baseline noise” was then computed as the standard deviation of the “spike-removed SomArchon trace”. “Spike amplitude” for each spike is calculated as the difference between the peak spike fluorescence and the lowest fluorescence value within three data points prior to the spike. To calculate the spike SBR for a given neuron, we divided the average spike amplitude of all spikes detected in the neuron by the “subthreshold baseline noise” of the neuron.

### Subthreshold Vm calculation and DBS-induced Vm change estimation

Due to the variability of the absolute SomArchon fluorescence values across neurons, we computed the subthreshold Vm trace for each neuron by dividing its “spike-removed SomArchon trace” by the average “spike amplitude” of that neuron. In addition, for Figs. 2, 3, 5, we computed the normalized Vm for each neuron by subtracting the mean Vm fluorescence of the given neuron during the 1 second pre-stimulation baseline period immediately before DBS onset. The population DBS-induced Vm change was then computed by averaging across all neurons. Similarly, for Fig. 6. The normalized Vm and firing rate for each neuron was computed by subtracting the pre-Opto baseline, and the population response to DBS was computed across all neurons.

### Spectral decomposition

Spectral decomposition of SomArchon Vm was performed with the FieldTrip Matlab toolbox^73^ (https://www.fieldtriptoolbox.org/), using wavelet morlet functions (5 cycles, Fig. 2) or using a filtering approach (butterworth filter) combined with Hilbert Transform (Fig. 4). The complex wavelets coefficients or the analytical signals were computed by the Hilbert Transform, from which we derived the phases that were used to compute spike-Vm phase locking values. For spectral power, we used the complex wavelet coefficients only.

### Spike phase locking value (PLV) calculation

To obtain a measure of how consistent spikes occur relative to the phase of an oscillation we calculated the phase locking value^74^ (PLV), defined as:

1 $${PLV}\,\left(f\right)=\left|\frac{1}{N}\right|\mathop{\sum}\limits_{N}{e}^{i \emptyset (f,n)}$$

where the phase of a given frequency *f* was obtained from the Hilbert Transform of the subthreshold Vm or the optogenetic stimulation pulse train. Hilbert Transform was performed on filtered Vm or optogenetic stimulation train using a Butterworth filter (filter order = 2) computed at 2 Hz steps with ±2 Hz bandwidth. For the PLV estimation at the single neuron level, we only included neurons that had more than 5 spikes (Figs. 7, 8, Supplementary Fig. 3). For the population PLV (Fig. 4), spikes from all recorded neurons were concatenated. This allowed us to obtain robust PLV estimates despite low spike rates in baseline and post-DBS conditions.

### Reporting summary

Further information on research design is available in the Nature Portfolio Reporting Summary linked to this article.

## Supplementary information

Supplementary Information

Reporting Summary

## Data Availability

The experimental raw data that support the findings of this study are available from the lead contact upon request. The source data for the relevant statistics are provided with this paper and are available at the Gihub repository: https://github.com/HanLabBU/Lowet-Nature-Communication-2022. Source data are provided with this paper.

## Source data

Source Data

## Code availability

Codes used for data analysis are available on Github repository: https://github.com/HanLabBU/Lowet-Nature-Communication-2022. All scripts were written and executed with Matlab2019b. Any additional information required to reanalyze the data reported in this paper is available from the lead contact upon request.
