# Supplementary material for: Deep brain stimulation creates informational lesion through membrane depolarization in mouse hippocampus
Source: Nat Commun. 2022 Dec 13;13:7709. doi: 10.1038/s41467-022-35314-1 (PMC9748039; doi:10.1038/s41467-022-35314-1)
Supplement: Supplementary file 1 — Supplementary Information [file 41467_2022_35314_MOESM1_ESM.pdf]

Supplementary Figures

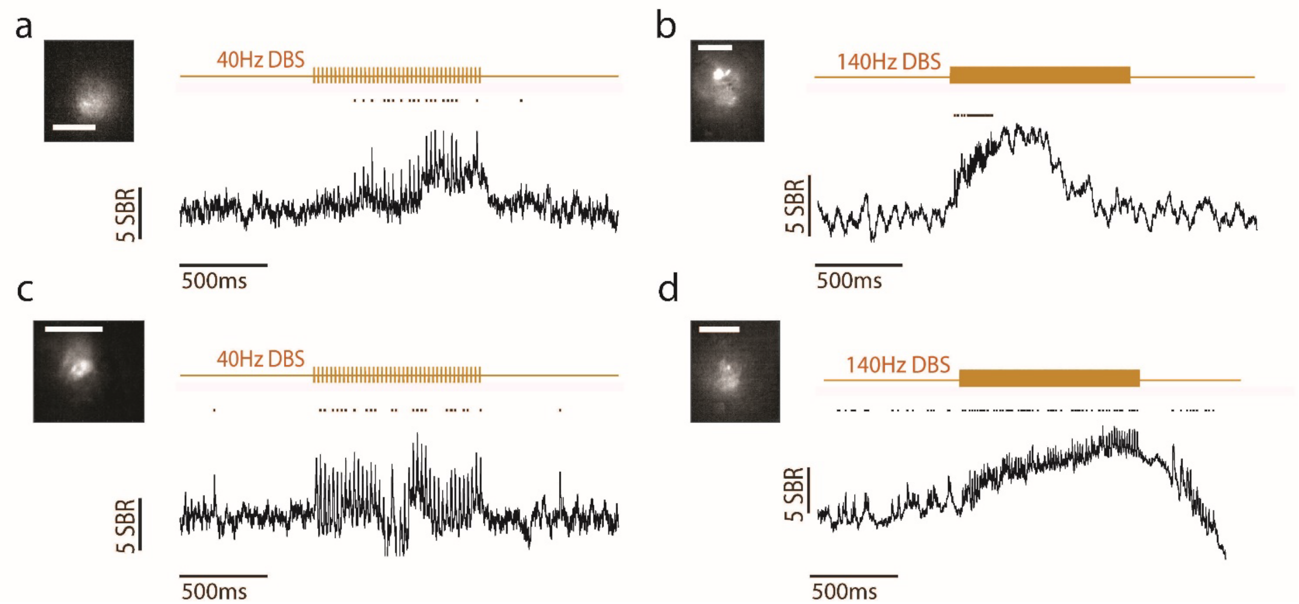

Supplementary Figure 1. *(a). Example SomArchon fluorescence before, during and after 40Hz DBS. Average SomArchon fluorescence of an example neuron is shown in the left upper image. Scale bar, 15µm. SomArchon trace is shown in black, detected spikes are marked by black ticks and electrical stimulation pulse patterns are in gold. SBR is the spike-to-noise ratio, which is defined by the spike amplitude divided by the standard deviation of the subthreshold slow SomArchon trace fluctuations (see methods). (b). Same as (a), but for 140Hz DBS.(c-d) Same as (a-b), but other example traces.*

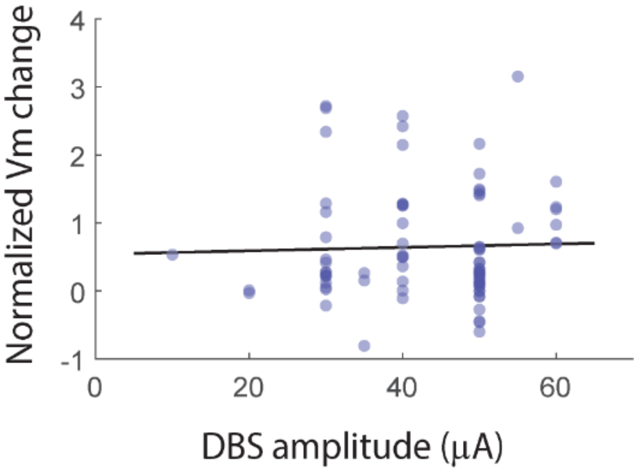

Supplementary Figure 2. *The amount of membrane voltage change during DBS for each neuron (n=83, dots) is shown as a function of DBS current amplitude used for the recording. Vm change is normalized bythe average spike amplitude for each recorded neuron. The black line shows the fitted linear regression line. All recording sessions are included, with and without optogenetics. Source data are provided as a Source Data file.*

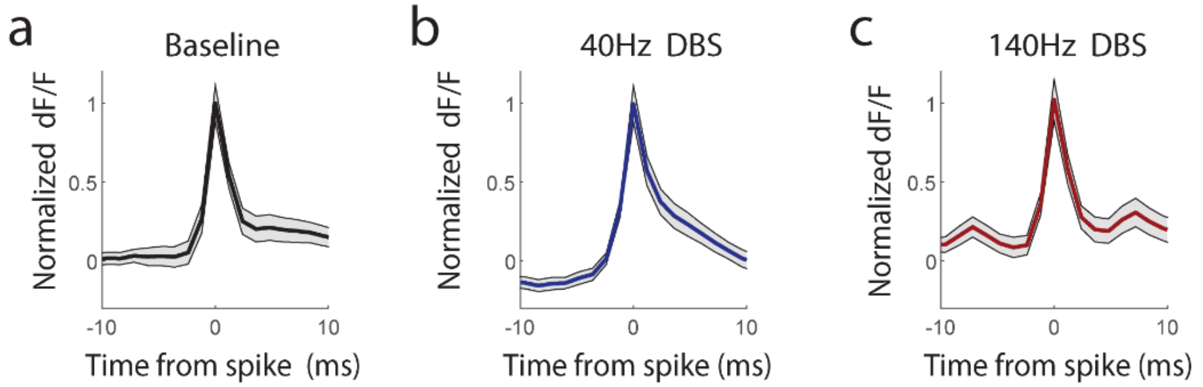

Supplementary Figure 3. *(a). Normalized SomArchon fluorescence trace aligned to spike peak during the baseline period (the 1 second time window before DBS onset). SomArchon fluorescence for each neuron is obtained by first dividing by the averaged spike amplitude of the neuron and then subtracting the mean fluorescence during the -72ms to -12ms (50 frames) before spike peak. The center thick line is the mean SomArchon fluorescence across neurons, and the shading outlined with two thin black lines are ±standard error (n=21 neurons). (b). Same as (a), but for spikes occurring during 40Hz DBS. (c). Same as (a), but for spikes occurring during 140Hz DBS (n=26 neurons).*

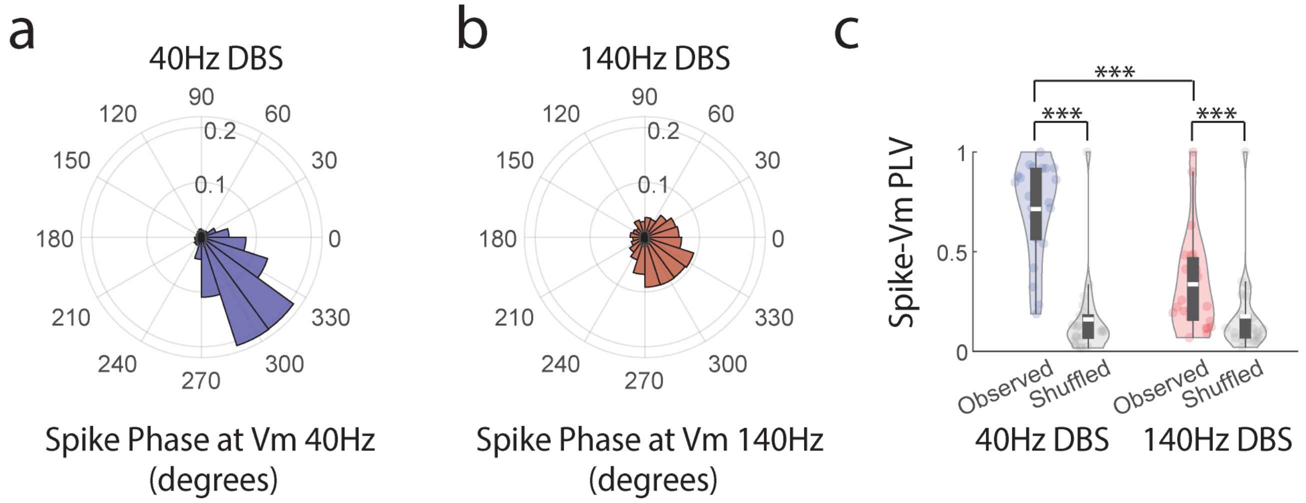

Supplementary Figure 4. *(a). The population polar histogram of spike times relative to the phase of Vm filtered at 40Hz in an example neuron during 40Hz DBS. Vm 40Hz oscillation phase was estimated from morlet wavelets (5 cycles). (b). Same as (a), but for an example neuron during 140Hz DBS. (c). Quantification of the spike phase-locking values (PLV) relative to Vm during 40Hz or 140Hz DBS. To determine whether an observed PLV is significantly above a random distribution, we shuffled the spike times relative to the recorded Vm to obtain a PLV null distribution. Data are visualized as violin plots with the outer shape representing the data kernel density and a blox plot showing interquartile range (1x, 1.5x). The white line is the mean. Two-sided paired t-test for within DBS condition statistics and two-sided independent t-test for between DBS condition statistics. Only neurons with >5spikes were included. 40Hz DBS, paired t-test, DBS vs shuffled data, df=19, p=5.77 x10^{-8}. 140Hz DBS, paired t-test, DBS vs shuffled data, p=2.1 x10^{-4}. Comparison between 40Hz and 140Hz DBS, independent t-test, p= 6.5 x10^{-6}, df=41. ***<0.001. Source data are provided as a Source Data file.*

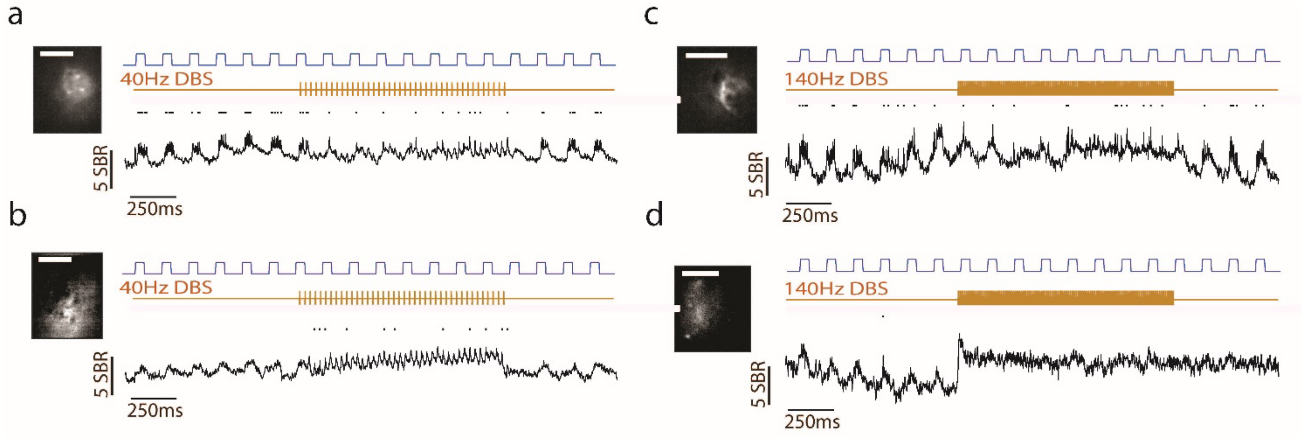

Supplementary Figure 5. *(a). An example CA1 neuron's SomArchon fluorescence trace (black) and spikes (black ticks) during 8Hz CoChR activation (blue line) and 40Hz DBS (gold line). 8Hz CoChR activation occurred throughout the 3-seocnd trial, whereas 40Hz DBS occurred for 1 second in the middle of each trial. (b) Same as (a), but another example trial of another neuron. (c-d) Same as (a-b), but example single-trial recordings from two additional neurons during 140Hz DBS.*
